# Race to the Top: evaluation of a novel performance-based financing initiative to promote healthcare delivery in rural Rwanda

**DOI:** 10.3402/gha.v9.32943

**Published:** 2016-11-28

**Authors:** Evrard Nahimana, Ryan McBain, Anatole Manzi, Hari Iyer, Alice Uwingabiye, Neil Gupta, Gerald Muzungu, Peter Drobac, Lisa R. Hirschhorn

**Affiliations:** 1 Partners In Health | Inshuti Mu Buzima, Kigali, Rwanda; 2 Department of Global Health and Social Medicine, Harvard Medical School, Boston, MA, USA; 3 Partners In Health, Boston, MA, USA; 4 Department of Epidemiology, Harvard School of Public Health, Boston, MA, USA; 5 Division of Global Health Equity, Brigham and Women’s Hospital, Boston, MA, USA; 6 Ministry of Local Affairs, Kigali, Rwanda; 7 Feinberg School of Medicine, Northwestern University, Chicago, IL, USA

**Keywords:** results-based financing, priority setting, capacity building, competition, Rwanda

## Abstract

**Background:**

Performance-based financing (PBF) has demonstrated a range of successes and failures in improving health outcomes across low- and middle-income countries. Evidence indicates that the success of PBF depends, in large part, on the model selected, in relation to a variety of contextual factors.

**Objective:**

Partners In Health∣Inshuti Mu Buzima aimed to evaluate health outcomes associated with a novel capacity-building model of PBF at health centers throughout Kirehe district, Rwanda.

**Design:**

Thirteen health centers in Kirehe district, which provide healthcare to a population of over 300,000 people, agreed to participate in a PBF initiative scheme that integrated data feedback, quality improvement coaching, peer-to-peer learning, and district-level priority setting. Health centers’ progress toward collectively agreed upon site-specific health targets was assessed every 6 months for 18 months. Incentives were awarded only when health centers met goals on all three priorities health centers had collectively agreed upon: 90% coverage of community-based health insurance, 70% contraceptive prevalence rate, and zero acute severe malnutrition cases. Improvement across all four time points and facilities was measured using mixed-effects linear regression.

**Findings:**

At 6-month follow-up, 4 of 13 health centers had met 1 target. At 12-month follow-up, 7 centers had met 1 target, and by 18-month follow-up, 6 centers had met 2 targets and 2 centers had met all 3. Average health center performance had improved significantly across the district for all three targets: mean insurance coverage increased from 68% at baseline to 93% (*p*<0.001); mean number of acute malnutrition cases in the previous 6 months declined from 24 to 5 per facility (*p*<0.001); and contraceptive prevalence increased from 42 to 59% (*p*<0.001). A number of innovative improvement initiatives were identified.

**Conclusion:**

The combining of PBF, district engagement/support, and peer-to-peer learning resulted in significant improvements despite resource constraints and is now being considered as a model for scale-up in other districts of Rwanda.

## Introduction

Over the past decade, Rwanda has made significant progress toward improving key health outcomes. Recent data show that the country is likely to have achieved all health-related Millennium Development Goals (1–4). To reach these milestones, Rwanda has focused in particular on strengthening primary health care delivery through decentralization of services to lower-level facilities, establishing a robust cadre of community health workers (CHWs), and development of community-based health insurance (CBHI) and performance-based financing (PBF) initiatives (3, 5, 6).

In 2006, PBF in Rwanda was scaled nationally at the district hospital level (7–9), with the goal of improving access and quality of health services (2). Under this model of PBF, payment was made directly to health facilities based on progress toward achieving pre-determined targets to incentivize health providers as well as reward successful health centers with resources for further improving infrastructure and operations. A number of studies have demonstrated positive impacts of this strategy, such as increased uptake of maternal and child health services (9–11).

While many low-income countries have adopted PBF strategies to link payment structures to quality and quantity of care, there is an ongoing debate on the sustainability, cost-effectiveness, and measurability of outcomes associated with PBF (12, 13). Most researchers, including those cited here (12–15), have concluded that the long-term success of PBF depends on an array of factors including: who receives incentives, the size and timing of incentives, selected indicators and targets, extent of technical support and mentorship to health providers and managers, and efforts to promote local ownership and leadership. Therefore, the way in which the program is designed – including its responsiveness to the local context – is critical.

Based on their results of a systematic review of PBF, Witter et al. (12) recommended that district-based initiatives with person-centered approaches should be designed to respond to local context, including regional disease burden and facility-level difficulties. This presents several key questions: How can PBF be strengthened to respond to local variation and priorities, increase value placed on processes, and promote a culture for learning and change? To the extent these questions can be answered, countries like Rwanda are positioned to achieve success not just on a national scale but also on a district-level scale in the poorest and most rural communities of the country.

Under Rwanda's existing PBF model, health facilities are evaluated on a set portfolio of nationally defined indicators. However, burden of disease and quality challenges vary significantly across districts (16). For example, the high prevalence of malaria in the Eastern Province differs remarkably from prevalence in the Northern Province (2, 16). In addition, the structure of PBF is focused narrowly on measuring and rewarding outcomes, without significant investment in the processes needed for improvement and achieving local ownership. The result is that facilities with poor outcomes are at risk of falling behind, while others succeed.

Over the past 10 years, Partners In Health (PIH) and its Rwandan counterpart, Inshuti Mu Buzima (IMB), have worked in partnership with the Ministry of Health to bring health care to Kirehe, one of the poorest, rural districts in Rwanda (17). In 2013, PIH and Kirehe district leadership introduced ‘Race to the Top’ (*Inkera y'Imihigo*), a district-wide PBF scheme to reward health centers upon reaching district-identified targets that reflect district-level health priorities, supported with technical support in quality improvement (QI) and peer-to-peer learning.

It was designed to innovate on the existing national PBF model, reflecting some of the recommendations from PBF studies. This included reflecting local challenges and priorities through stakeholder engagement throughout the process and in the targets chosen, and encouraging peer-to-peer learning through sharing of results and interventions and incorporation of QI capacity building. We describe the development and implementation of the Race to the Top (RTT) initiative, evaluate results over an 18-month timeframe, and discuss best practices and innovations made by participating health facilities.

## Methods

RTT (Inkera y Imihigo, in Kinyarwanda) was inspired by a US Department of Education initiative of the same name that offered incentives to states in order to encourage reforms within the education system toward improved teaching and learning (18). In 2013, PIH leadership, together with district-level authorities within the Ministry of Health, initiated RTT as a district-wide approach using PBF to encourage health providers to excel in clinical and support areas chosen as priorities by a range of district stakeholders. The program was first implemented in Kirehe district, which is home to 312,000 individuals, 3,500 community health workers, 13 health centers, and one district hospital. In 2014, three new health centers opened their doors and joined the initiative.

### Choosing targets

The planning phase of RTT began in October 2013 and involved a series of meetings with a range of stakeholders – including community leaders, district authorities, health center managers, hospital supervisors, and mentors. Through discussions, three main areas in the health sector were selected as priorities: 1) access to health care through community-based health insurance, 2) reduction of severe malnutrition for children under-five, and 3) increasing family planning uptake.

At the time of implementation, Kirehe was in the top five districts with the highest of global acute malnutrition in children aged 6–59 months. Access to care through health insurance was also among the lowest in the country. Finally, although family planning uptake in Kirehe was not different from that in other districts, stakeholders decided to include it because they felt its impact would be broad enough to influence other indicators as well.

Indicators to measure the priority areas were agreed upon and were given as follows:

*Family planning*: Contraceptive prevalence rate over the previous 6 months. The consensus target for this was 70% contraceptive prevalence. Data on progress were extracted from family planning registers, individual patient dossiers, community reports, and district-aggregated data. The denominator was the total number of women in reproductive age (15–49) living in each health center catchment area.

*CBHI*: Percentage of persons enrolled in CBHI. The target for this was 90%. Data were extracted from registers of beneficiaries as well as bank statements for individual financial contributions into CBHI. The denominator was the total population served by each health center.

*Child malnutrition*: Absolute number of children under-five who were diagnosed with severe acute malnutrition over the past 6 months. Facilities aimed to reduce this number to zero. Data were collected from active case finding reports, health center registers, hospital reports, and aggregated district reports.

Data were aggregated at 6-month intervals across four time points: baseline (0 months), 6-month follow-up, 12-month follow-up, and 18-month follow-up.

### PBF in RTT

**Fig. 1:**
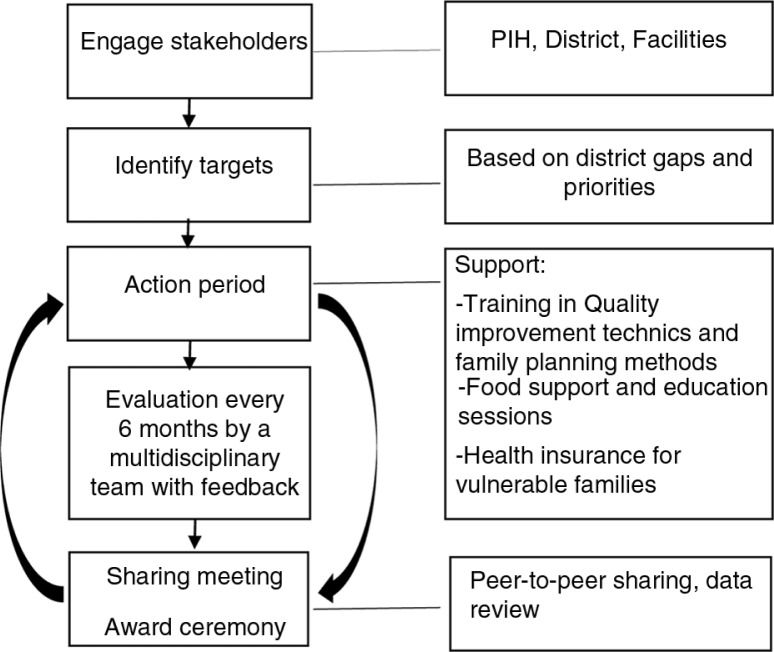
Race to the top implementation model.

There were three main components of RTT initiative (Fig. 1). First, there are facility-level financial payouts provided to the administration when they achieve the agreed upon targets. Second, there are bi-annual summits to share lessons learned, as well as successes and challenges, across facilities. Third, technical staff from the district hospital, district officers, and PIH employees provide routine mentorship on QI and support to facility administration.

Health centers were awarded extra funds only when they reached benchmarks toward all three targets. In order to foster positive competition among health centers, a ‘no loser model’ was employed. Specifically, every health center that reached all three targets would be awarded, rather than only the center with the highest or most rapid performance improvement. Payouts of approximately $5,000 were provided to the administration as one lump sum, and staff were required to invest this back in the facility in a manner of their choosing.

All facilities were provided trainings in QI methodology, as well as technical trainings in relevant domains of clinical delivery, upon request. Examples included long-acting reversible methods of contraception and detection of severe acute malnutrition cases.

During the action period between knowledge dissemination meetings, health centers developed and tested approaches to improving service delivery for family planning and nutrition, and for increasing uptake of CBHI. Examples of projects included the integration of nutritional cooking demonstrations with community-based screenings, community *tontines* (collective investment strategies) to increase CBHI adherence, and the creation of registry systems to link patients and CHWs for follow-up visits with malnourished children and pregnant women.

### Evaluation and sharing

Evaluation on progress was conducted by the evaluation team at the pre-determined intervals. The team was composed of hospital supervisors/mentors as well as district and PIH agents. They used standardized tools to extract the data from the sources noted above. Following data collection, the results were reviewed with the district monitoring and evaluation team to discuss any differences in routinely reported data to help improve data quality. Knowledge dissemination meetings attended by the teams from all health centers as well as stakeholders from the district were held every 6 months. The meetings included review of the results from all facilities as well as sharing for successful interventions (captured by the evaluation team) followed by award ceremonies. Following the analysis of change in performance at 18 months, the team held discussions with stakeholders to help understand the contributing factors associated with changes observed.

### Data analysis

A mixed-effects linear regression framework was used to examine the magnitude of change over time for each of the areas targeted. The application of linear regression with binary outcomes, along with robust standard errors, allows for the interpretation of coefficients as percentages. The primary coefficient of interest was the effect of time, while the inclusion of facilities as fixed effects allowed for comparisons in outcomes across sites. A random effect accounted for clustering of individual observations within facilities over time. The mode of analysis was intention-to-treat, meaning that facility-level data were included even if no data were available at specific time points. This study received authorization of the Rwandan National Ethics Committee, a Ministry of Health's Institutional Review Board.

## Results

We describe results from 13 health centers which participated when the initiative was launched in December 2013 (three new health centers were opened and joined the initiative after the start of RTT) (Table 1). At baseline, the mean contraceptive prevalence across facilities was 42.4% (SD=11.4%), mean percentage of the catchment population enrolled in CBHI was 68.3% (SD=15.6%), and the average number of severe acute malnutrition cases for the under-five population over the past 6 months (incident cases) was 24.0 (SD=16.2) (see Table 2 for more details).

**Table 1:** Characteristics of health centers at baseline (December 2013)

Facility	Catchment population	Annual patient volume	CBHI coverage (%)	Contraceptive prevalence (%)	Severe acute malnutrition cases
Bukora	25,372	47,685	72.3	40.7	45
Gahara	29,704	36,709	93.0	32.8	24
Gashongora	17,946	36,853	61.0	33.8	16
Kabuye	9,933	22,442	72.0	57.4	10
Kirehe	63,013	80,511	59.8	41.0	29
Mulindi	18,428	27,971	58.5	46.8	4
Musaza	26,610	50,696	62.3	56.6	13
Mushikiri	18,810	31,070	94.9	23.2	30
Nasho	19,996	32,507	76.0	44.3	0
Ntaruka	17,327	13,239	84.3	55.3	25
Nyabitare	10,106	22,748	43.3	45.7	48
Nyamugali	36,511	70,746	59.0	23.8	50
Nyarubuye	18,679	41,573	51.0	49.5	18
Total	312,435	514,750	68.3	42.4	24

Annual patient volume based on aggregate facility data from 2013. CBHI, community-based health insurance.

**Table 2:** Change over time in three RTT indicators

	Baseline	6 months	12 months	18 months	Total change	B (95%CI)	*p*
CBHI coverage	68.3%	73.3%	87.3%	93.1%	24.9%	0.248 (0.181, 0.314)	*p*<0.001
Contraceptive coverage	42.4%	43.6%	46.9%	59.2%	16.8%	0.206 (0.126, 0.276)	*p*<0.001
Malnutrition cases	24.0	23.6	27.7	6.5	17.5	17.57 (−28.70, −6.42)	*p*=0.002

CBHI, community-based health insurance; RTT, Race to the Top.

### Contraceptive prevalence

Contraceptive prevalence increased by 1.2 percentage points from 0 to 6 months (β=0.062; *p*<0.05). The overall increase at 12 months and at 18 months was 4.5% (β=0.095; *p*<0.001) and 16.8% (β=0.218; *p*<0.001) respectively. Change in prevalence varied from an increase of 46.5% at Nyamugari to a decrease of 8.6% at Nyabitare over the full 18-month period.

### Community-based health insurance

From 0 to 6 months, insurance enrollment increased 5.0% across facilities, which was non-significant (β=0.050; *p*=0.15). At 12 months, enrollment had increased 19.0% compared to baseline (β=0.189; *p*<0.001), and 24.9% points total by the end of the study (β=0.248; *p*<0.001). Change in enrollment over the study period ranged from a 44.0% increase at Nyarubuye to a 2.9% decrease at Mushikiri. Overall, by 18 months, all 13 facilities achieved the target 90% coverage of CBHI.

### Child malnutrition

The absolute number of malnutrition cases decreased, on average, by 0.4 cases from baseline to 6-month follow-up (β=−0.38; *p*=0.95), 3.7 cases from baseline to 12-month follow-up (β=3.67; *p*=0.52), and 17.6 cases (β=−17.59; *p*=0.002) from baseline to 18-month follow-up. Change in total malnutrition cases over the study period ranged from a decrease of 48 cases in Nyabitare to an increase of 3 cases in Nasho. In total, 4 of 13 facilities (30.7%) achieved the target of zero malnutrition cases.

## Discussion

We assessed results from a novel PBF initiative at 13 health centers in Rwanda's rural Kirehe district, designed to strengthen local ownership and reflect local needs. Over the 18-month implementation period, health centers collectively demonstrated remarkable progress, improving their performance on all three objectives. The mean percent of CBHI coverage increased from 68 to 93% and of contraceptive prevalence improved from 42 to 59%, and malnutrition cases declined from an average of 24 cases per facility to an average of 5 cases per facility. The innovation in this model of PBF included a strong emphasis on local engagement and leadership in determining the priority areas, supporting peer-to-peer learning and supporting and celebrating capacity and local QI initiatives to address identified gaps (Table 3). We believe that these three components were important in contributing to the quantitative success we measured.

**Table 3:** Activities and innovations for achieving Race to the Top objectives

Domain	Examples of activities and innovations
Increase community-based health insurance	• Community tontines (investment plans) to increase health insurance coverage
	• Campaigns to reach remote communities
	• Subsidized payments for vulnerable families
	• Partnership and collaboration with local authorities
	• Electronic databases for tracking community members
Increase uptake of contraception	• Active collaboration between local authorities and community health workers
	• Systematic assignment of community health worker to females within a specified catchment area
	• Active case finding of lost to follow-up patients
	• Integration of family planning across all health center services
	• Availability of family planning services 7 days a week
	• Use of quality improvement techniques such as PDSA cycles
	• Outreach visits for women in remote areas
Reduce severe acute malnutrition	• Integration of cooking demonstrations, severe acute malnutrition screenings and food support distribution within local communities
	• Active case finding of lost to follow-up children with severe acute malnutrition
	• Provision of livestock for extremely vulnerable families
	• Monthly provision of fish for families with children under-five
	• Electronic data base for patient tracking of children under-five with severe acute malnutrition

PDSA (Plan, Do, Study, Act).

### Community and leadership engagement

Unlike many health delivery projects in which outside donors or national policies drive the priority setting process (19), local district officials, health centers, and community representatives assumed responsibility and led the activities of the RTT initiative, including the selection of indicators and targets. At the outset of the initiative, the mayor remarked that RTT ‘should be an opportunity for all of *us* to address *our* district health priorities’. The mayor appointed an independent evaluation committee, composed of three hospital mentors, the PIH clinical directorship, and members of the health district administrative unit. Every 6 months’ progress was evaluated, and health centers convened a joint knowledge dissemination meeting.

There are numerous examples in which local community participation in health care delivery has had a positive impact on outcomes, ranging from increased adherence to antiretroviral therapy to reduced infection rates of malaria (20–22). However, there is less evidence on the impact of local leadership in priority setting compared to top-down national mandates. Available evidence on the relationship between local and national priority setting and engagement largely comes from high-income countries (23). One major exception to this is the African Health Initiative Population Health Implementation and Training (PHIT) partnership across several sub-Saharan African countries, of which this project is a part (24). In the current study, we found that members of the community and staff routinely reported a sense of ownership and active involvement in ensuring its success. Participants also reported that this local participation and ownership in priority setting was pivotal for the community buy-in and continued success at 18 months of the RTT initiative.

### Peer-to-peer learning

Within the context of global health delivery, peer-to-peer learning is considered a best practice for promoting QI as well as in operational research (25). For example, modifying implementation design of a program to train health personnel by incorporating peer-to-peer learning significantly increased the adoption of essential birth practices in Uttar Pradesh, India (26). In the context of RTT, peer-to-peer learning was facilitated through two modes: first, at knowledge dissemination meetings held every 6 months, representatives from all 15 participating health centers gathered to share progress on program indicators and targets. Best performers were asked to present on strategies they had implemented to achieve targets, and underperforming sites presented challenges for peer feedback and reflection.

Second, ‘learning visits’ were held at sites demonstrating exemplary improvement, in which representatives from other sites had the opportunity to directly observe real-time implementation. For example, at the outset of RTT, Gashongora health center implemented a novel model for improving family planning, and hosted representatives from Mahama and Kigarama health centers to observe this model in action. Both health centers subsequently adopted the approach.

### QI and local innovation combined with data feedback

The need for QI projects with a fixed 6-month timeframe stimulated innovation and fostered excitement among staff, as well as providing fodder for discussion at knowledge dissemination meetings. While projects at some health centers were successful, and others less so, those that were the most innovative were readily adopted by other sites, as all sites were motivated to achieve targets and receive payouts tied to the PBF model. These activities were facilitated by baseline training in QI methods as well as mentoring by the visiting teams to encourage and provide assistance as needed.

Our approach differs from a number of other PBF initiatives, which typically have established national targets which may not represent local gaps and do not incorporate as strong an emphasis on building and supporting the local innovation and sharing for improvement (9–12). A broad base of research and experience underscores the centrality of QI projects in ensuring effective health care delivery (27). Our findings support the linkage of project outcomes to financial incentives supported by QI capacity building and creating a community of sharing rather than solely competition as an effective strategy for promoting collaboration across sites, particularly under the ‘no loser’ framework for payouts. Challenges to the implementation included the lack of engagement and strong leadership in some health centers which required greater investment in mentoring and slowed some progress. The project also overlapped in time when there was a large influx of individuals into the district which created an unplanned increase in need for CBHI, family planning, and nutritional support, requiring increased effort to achieve the targets (28).

### Limitations

The study presents some limitations: first, RTT is a local initiative whose main aim is to respond to district-wide specific health priorities with specific indicators which were developed in partnership across stakeholders and included data validation by the evaluation team. Therefore, we couldn't randomize facilities to measure impact or compare change in these indicators in other districts, which limits our ability to ascribe improvements to the intervention alone. However, because of the close engagement of stakeholders, the team was aware of other work in the district, and no large interventions targeting these areas outside of RTT were identified during the period reported. Second, PBF schemes are inherently subject to data quality challenges, particularly sites that may over- or under-report outcomes to meet targets. We tried to minimize this by having the evaluation team comprised of a range of stakeholders who validated the measurement used to determine eligibility for the PBF. Third, the movements of populations within and outside the district may have resulted in changes in the estimation of the eligible population particularly for the family planning and community health insurance indicators. Finally, we did not do a formal mixed methods study, and so our hypothesis of the three components that made this successful PBF intervention will need further validation in efforts to spread to other districts in Rwanda and the region.

## Conclusion

In summary, the RTT initiative – as a form of locally driven PBF – was associated with significant change in the targeted areas of child severe acute malnutrition, family planning, and health insurance adoption. Importantly, the initiative resulted in a large number of locally designed and implemented improvement projects and evidence of spread of effective approaches within the district. The success of implementation of the initiative was related to a high level of self-reported commitment to ensure local engagement and leadership, peer-to-peer learning, and capacity and support for QI and innovation. Moving forward, we plan to partner with the Ministry of Health in applying the lessons learned from RTT and extend this initiative to other districts of Rwanda.

## Paper context

Performance-based financing has been implemented in Rwanda and in other low-limited resource settings with various health-related outcomes. We implemented Race to the Top as an innovation model that combines community engagement for priority setting, performance-based awards, and technical coaching to provide opportunity for improvement to health providers in rural Rwanda. Evaluation of the intervention showed improvements to respond to local priorities through innovations, team work, peer learning, data use, and feedback.
